# MICRO (Multimaterial, Integrated, Compact, Ready-to-Plug, One-Step 3D-Printed): A Simple-to-Use Electrochemical Device for On-Site Analysis and Drug Screening

**DOI:** 10.1021/acsomega.5c06692

**Published:** 2026-02-16

**Authors:** Tiago Moraes Zavarize, Fárlon Felipe Silva Xavier, Augusto dos Santos Novais, Celso Luciano de Araújo, Edmar Isaias de Melo, Ettore Ferrari Júnior, Bruno Gabriel Lucca, Lucas Franco Ferreira, Rodrigo Amorim Bezerra da Silva

**Affiliations:** † Institute of Chemistry, 28119 Federal University of Uberlândia, Uberlândia, Minas Gerais CEP 38400-902, Brazil; ‡ Institute of Chemistry, 28119 Federal University of Uberlândia, Monte Carmelo, Minas Gerais 38500-000, Brazil; § Forensic Analysis Laboratory, Criminalistic Institute, Civil Police of the Federal District, Federal District, Brasília 70610-907, Brazil; ∥ Institute of Chemistry, 54534 Federal University of Mato Grosso Do Sul, Campo Grande, Mato Grosso Do Sul 79074-460, Brazil; ⊥ Institute of Science and Technology, 74380 Federal University of the Jequitinhonha and Mucuri Valleys, Diamantina, Minas Gerais 39100-000, Brazil

## Abstract

3D printing has emerged as a transformative technology in electroanalytical chemistry, enabling the rapid, low-cost, and reproducible mass fabrication of portable devices and sensors for several areas such as environmental, clinical, food, and forensic applications. Herein, we present a multimaterial, integrated, compact, ready-to-plug, and one-step additively manufactured electrochemical cell (MICRO-EC^3D^). This all-in-one device was fabricated using a dual-extruder 3D printer and comprises a single unit containing the three electrodes/tracks (conductive carbon black–polylactic acid, CB-PLA) as well as the body containing an embedded mini solution reservoir (polyethylene terephthalate glycol, PETG). This arrangement eliminates the assembling of external accessories and allows the introduction of miniaturized stirrers. Unlike previous similar 3D-printed devices, the proposed microcell is readily interfaced to mobile apparatus (hand-held potentiostats and smartphones) through user-friendly USB-based connectors (wireless, crocodile clip, or banana pin). After a quick chemical (immersion in 5.0 M NaOH for 3 min) and electrochemical treatment (+1.4 V for 200 s and −1.0 V for 200 s in 0.5 M NaOH), this device showed performance comparable to a commercial carbon screen-printed electrode for model analytes (ferricyanide, paracetamol, and nitrite). The analytical use of the device was demonstrated for the square-wave voltammetric detection of anabolic steroid oxymetholone (OXM), in which two linear responses (1.0 to 5.0 and 6.0 to 10.0 μM; *R* ^2^ > 0.99) and a limit of detection of 0.21 μM were obtained. These values were suitable for the screening of OXM in three seized samples, yielding results consistent with gas chromatography–mass spectrometry. These findings evidence the potential of MICRO-EC^3D^ as an easy-to-use, portable, and low-cost (<ten cents) tool for on-site preliminary forensic analysis and for increasing the acceptance and popularity of electrochemical methods, mainly among inexpert analysts.

## Graphical abstract

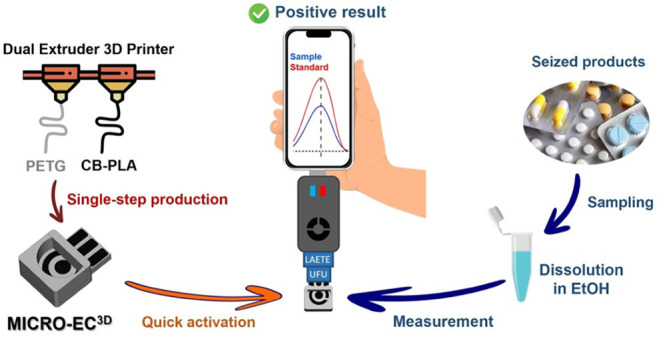

## Introduction

Electroanalytical chemistry is an emerging field that has been offering alternative methods for qualitative and quantitative analyses, providing simpler, more sensitive, portable, and cost-effective approaches compared with conventional analytical techniques employed across various knowledge sectors. However, traditional electrochemical setups (body cell and commercial electrodes) present typical drawbacks such as (a) high cost, (b) weakness of glass cells, (c) higher dimensions of the cell (volumes in milliliter order), (d) difficulty in operation, assembly, and right connection of electrodes with the potentiostat, and (e) the need for cleaning the working electrode between measurements.^1^ To overcome these problems, three-electrode integrated electrochemical cells (TEI-ECs) or “all-in-one” devices such as screen-printed electrodes (SPEs) have been marketed and are better accepted by users overall.^2,3^ SPEs provide the benefits of being miniaturized (waste in microliter order), disposable, versatile, portable, reproducible, ready-to-use, and user-friendly.^4^ Thus, they have been used to develop on-site methods for healthcare diagnostics, pharmaceutical (e.g., quality control), environmental monitoring, food and drink analysis, and forensic (e.g., drug detection).^4–6^ However, most commercial SPEs are expensive, especially in countries outside the US and Europe (e.g., ≈five dollars per device in Brazil).

Among alternative techniques to produce cheaper TEI-EC, 2D-technologies such as lab-made screen printing,^6^ laser engraving,^7^ and 3D printing (additive manufacturing, AM) by fused filament fabrication (FFF) are in the spotlight due to affordable costs and automated mass production.^6^ Nevertheless, FFF (layer-by-layer deposition of polymeric filaments) is more versatile since it allows the creation of devices in a myriad of 3D shapes (“freedom of design”) and the production of bespoke hybrid devices through the combination of several available polymers (e.g., resistant, flexible, translucent, conductive).^8–11^ Moreover, considering the rapid expansion of the 3D printing market, improved printers (multimaterial, faster, and easier-to-use) tend to boost the creation of innovative electroanalytical devices. In addition, thermoplastic polymers are recyclable, promoting more sustainable analysis and circular economy electrochemistry.^12^

Some research groups have proposed TEI-EC through AM (FFF). In the current state, different strategies have been used to produce substrates and electrodes (insulating and conductive filaments, respectively): (a) individual printing of the cell and electrodes followed by the components’ assembly;^13–18^ (b) manufacture of a template using a 3D printer or day-to-day materials and production of patterned electrodes using a 3D printing pen;^19–23^ and (c) single-step fabrication of hybrid devices (substrate and electrodes) in a multimaterial single nozzle 3D printer (filament change during production)^24–26^ or dual-extruder printer (two filaments printed in independent nozzles).^27–33^ The last approach (c) is advantageous as it allows the automated production of TEI-EC and does not require the assembly of the cell components in most cases.^9,10^ Although interesting, most of these devices mimic those commercially available and exhibit impractical electrical connections (e.g., using wires, crocodile clips).^11^ These features limit their use and diminish the acceptance and popularization of electrochemical methods by general users (e.g., forensic experts, health professionals).

In this context, a Miniaturized, Integrated, Cheap, Ready-to-plug, and One-Step produced 3D-printed electrochemical cell (MICRO-EC^3D^) is proposed as a novel tool for more convenient electroanalysis and forensic applications. This hybrid microdevice was fabricated using a low-cost dual-extruder 3D printer, which prints the body containing an embedded solution reservoir and electrodes at independent nozzles using polyethylene glycol terephthalic acid (PETG) and carbon black-polylactic acid (CB-PLA), respectively. To ensure practicality and versatility, MICRO-EC^3D^ contains three USB-based connectors for its use with different potentiostats (wireless, banana plug, and crocodile clip) and may be used with a small lab-made 3D-printed stirrer, which is required for hydrodynamic electrochemical measurements. The electrochemical performance of ferricyanide was evaluated before and after chemical and electrochemical activation in NaOH. Moreover, the device responses in the presence of model analytes (ferricyanide, paracetamol, and nitrite) were compared with those obtained at a commercial carbon screen-printed electrode (C-SPE). Lastly, the voltammetric screening of oxymetholone (OXM) in seized samples was performed to demonstrate the analytical feasibility of MICRO-EC^3D^ for on-site forensic analysis.

OXM is an anabolic androgenic steroid (AAS) developed in 1959 to treat anemia and osteoporosis and to promote muscle growth in conditions such as muscular atrophy.^34^ Because of its potent anabolic effects, this compound has been misused by both professional and amateur athletes, as it is on the list of definitive prohibited substances of the World Anti-Doping Agency (WADA).^35^ In Brazil, the marketing of formulations containing OXM has been prohibited by the National Health Surveillance Agency (ANVISA) since 2017.^36^ Consequently, the incidence of counterfeit and smuggled formulations labeled as OXM in the black market has increased in recent years.^37^ These samples often lack the active ingredient, are adulterated with other substances, or contain concentration levels lower than those indicated on the label.^37^ For the analysis of AAS in seized samples, a recommended method is gas chromatography–mass spectrometry (GC–MS),^38^ which requires large and expensive instrumentation. Thus, the development of quick, low-cost, and portable analytical methods for the on-site detection of OXM is relevant in the forensic field.

## Experimental Section

### Reagents and Solutions

The solutions were prepared in deionized water (resistivity >18 MΩ cm) using a Gehaka water purification system (São Paulo, Brazil) and analytical grade reagents. Glacial acetic acid, sodium acetate, boric acid, sodium dihydrogen phosphate, potassium chloride, sodium hydroxide, potassium ferricyanide (III), sodium nitrite, and ethanol were purchased from A.C.S. (São Paulo, Brazil) and paracetamol (PAR) from Sigma-Aldrich (Missouri, USA). Methandrostenolone and OXM reference material were prepared from the seized samples after characterization using Fourier-transform infrared (FTIR) spectroscopy and quadrupole time-of-flight liquid chromatography/mass spectrometry (QTOF/LC–MS). Seized OXM samples (tablets and capsules) were provided by the Criminalistics Institute of the Civil Police of the Federal District (PC-DF, Brazil). PAR and OXM solutions were prepared by dissolving the powder in ethanol and suitably diluted in an electrolyte for electrochemical measurements.

### Instruments, Apparatus, and Measurements

Electrochemical measurements were performed using hand-held potentiostats (Sensit Smart, EmStat 3 Blue, and EmStat 4 Low Range) from PalmSens (Houten, Netherlands), which are controlled by PSTrace 5.9 software (laptop) or PStouch app (smartphone). The measurements were performed in the presence of dissolved oxygen using the proposed MICRO-EC^3D^ and a commercial carbon SPE from DropSens (Oviedo, Spain) at room temperature for comparison. The WE of both devices exhibits the same geometric area (0.1256 cm^2^; Ø = 4 mm).

Electrochemical impedance spectroscopic (EIS) measurements were carried out for the electrochemical characterization of MICRO-EC^3D^. The following parameters were used: a frequency range between 1 Hz and 10 kHz, 10 data points per frequency decade, and the half-wave potential for the applied AC voltage. Scanning electron microscopy (SEM) images were acquired by a Tescan (Vega 3) instrument.

All 3D-printed objects (MICRO-EC^3D^, body of the mini lab-made stirrer, and body of connectors) were designed using FreeCAD software (STL file), sliced using Ultimaker Cura software (GCODE file), and printed using a Sovol SV04 independent dual extruder (IDEX) 3D printer (Sovol Technology, Shenzhen, Guangdong, China) equipped with two 0.4 mm nozzles (multimaterial printer). Insulating filaments (PLA and PETG) were purchased from 3D Fila (Belo Horizonte, Brazil), and conductive filament (CB-PLA) was purchased from Protopasta (ProtoPlant, Vancouver, USA). For the printing of PETG (nozzle 1), the following parameters were used: layer height of 0.2 mm, infill of 10%, speed of 60 mm s^–1^, nozzle temperature of 245 °C, and bed temperature of 85 °C. The same conditions were used for CB-PLA printing (nozzle 2), except for the infill (100%) and nozzle temperature (220 °C).

### Design and Fabrication of MICRO-EC^3D^

Figure 1A shows the production steps of MICRO-EC^3D^. The proposed device consists of two complementary designed pieces: (1) the body containing the embedded solution reservoir and (2) three electrodes (working electrode, WE; pseudoreference electrode, PRE; and counter electrode, CE) with respective tracks (electrical contacts). Using the slicer software, the objects were merged and sliced using the automated programming named as “dual mode”. This mode alternately extrudes different materials at two independent nozzles (PETG at nozzle 1 and CB-PLA at nozzle 2) and wastes less filament than a multimaterial single nozzle 3D printer. Video S1 (Supporting Information) shows the speed up printing operation of one device (∼8 min), and Figure S1 shows the images during and after the printing of a set of five devices. Optionally, PRE may be painted with a lab-made silver ink (silver powder/colorless nail polish at a ratio of 60/40 wt %), which dries after 30 min at room temperature. Figure 1B shows real images of MICRO-EC^3D^ with dimensions of 1.9 cm × 1.4 cm × 0.7 cm. As shown, the contact edges of both polymers are well-defined. This appearance contrasts with that observed in some recent single-step-produced TEI-EC, which had “blurred” edges.^26,31,32^ This worst resolution was caused by the multilayer printing of each polymer, which caused the simultaneous fusion of both polymers at the edges during the repeated passage of the hot nozzle. Thus, our device presented better definition due to the printing of a single layer of CB-PLA (Video S1). It is also important to mention that the connection length (distance from the beginning of the printed trails to electrodes) of 0.9 cm was the shortest value that promoted the connection with the metallic tracks from USB connectors. This minimal value was designed to decrease the electrical resistance and improve the electrochemical responses.^39^

**Figure 1:**
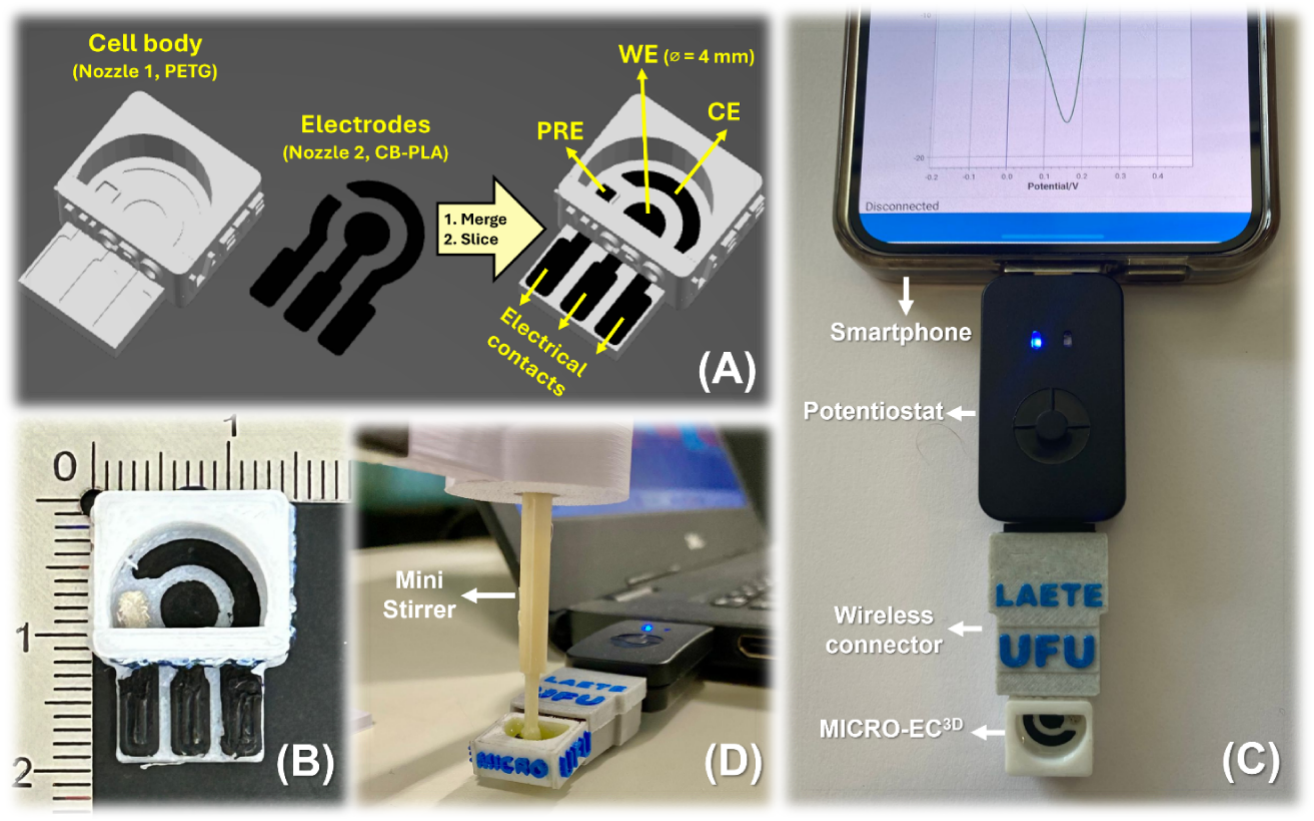
MICRO-EC^3D^ fabrication and use: scheme of sliced parts for 3D printing (A); top image of the 3D-printed cell (B); images of the wireless portable system: device interfaced to the SensitSmart potentiostat through the custom connector and controlled by a (C) smartphone or (D) laptop (including the mini stirrer). This version of MICRO-EC^3D^ contains the logo of our research laboratory (LAETE: Laboratory of Analytics, Electrochemistry, and Treatment of Effluents) and university (UFU: Universidade Federal de Uberlândia).

Figure 1C shows an image of the fully compact system composed of the microcell connected to a tiny potentiostat (Sensit Smart, PalmSens) and a smartphone. Typically, this potentiostat allows a simple electrical connection to several commercial SPEs (thinner than MICRO-EC^3D^). Therefore, we developed a wireless connector (Figure 1C and Figure S2A) to enable simple contact between the MICRO-EC^3D^ and the potentiostat (USB plug tracks fused to SPE tracks). Two other USB-based connectors may also be used to plug the microcell and potentiostats that use wires for electrical contact: 2 mm banana pins (Figure S2B) and crocodile clips (Figure S2C). Additionally, a mini lab-made 3D-printed mechanical stirrer set (Figure 1D and Figure S3) was developed for the control of the movement of solution into the reservoir. This set speeds up solution homogenization and is required to perform hydrodynamic measurements. The stirrer may be switched on/off (I/O) and operated at different stirring rates (Figure S3). Moreover, it is considerably portable, once it is fed by a 5.0 V power supply (e.g., a USB-port from a laptop).

## Results and Discussion

### Characterization of MICRO-EC^3D^

It is well-known in literature that the electrochemical performance of 3D-printed electrodes using commercial CB-PLA (Protopasta) is poor due to the low proportion of conductive particles in the composite (≈ 20 wt %), which requires activation procedure(s) to partially remove the PLA from the electrode surface (e.g., physical, chemical, electrochemical, biological, laser ablation^40^). Among these, an electrochemical treatment (ET: +1.4 V for 200 s and −1.0 V for 200 s in 0.5 M NaOH^13^) has been widely used due to its efficiency, simplicity, low toxicity, and lack of need for additional apparatus. In our case, the three CB-PLA electrodes of MICRO-EC^3D^ were simultaneously activated (short-circuited with the WE cable and using external Ag/AgCl/KCl_sat_ and Pt as RE and CE, respectively), as used elsewhere.^19,20^ This procedure produced better responses than the activation of the WE (CB-PLA) using the printed PRE and CE from the device, as suggested in the literature.^29^ To evaluate the electrochemical performance before and after this ET, cyclic voltammetry (CV) measurements were performed in the presence of 1.0 mM K_3_Fe­(CN)_6_ and 0.5 M KCl. For this study and electrochemical characterization in ferricyanide, CB-PLA was used as PRE. As shown in Figure 2, no faradaic peaks were observed without treatment (WT), and an ill-defined voltammetric profile (Δ*E*_p_ = 876 mV and *i*_pa_/*i*_pc_ = 0.75) was obtained after this ET. Thus, two chemical treatments (CTs) were investigated through the previous immersion of the device in NaOH solutions for 3 min (CT 1:0.5 M NaOH and CT 2:5.0 M NaOH). These treatments were investigated either alone or in combination with ET.

**Figure 2:**
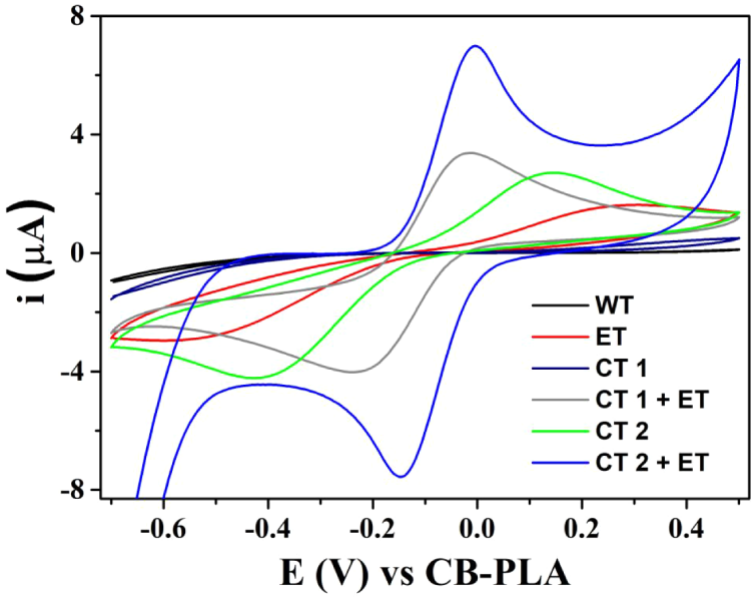
Cyclic voltammograms of MICRO-EC^3D^ in 1.0 mM ferricyanide and 0.5 M KCl without treatment (WT), with electrochemical treatment (ET: +1.4 V for 200 s and −1.0 V for 200 s in 0.5 M NaOH), chemical treatments (CT 1:0.5 M NaOH for 3 min and CT 2:5.0 M NaOH for 3 min), and combination of treatments. Scan rate: 50 mV s^–1^.

As presented in Figure 2, a poor voltammetric response was observed after CT 1. After CT 2, faradaic peaks began to appear (Δ*E*_p_ = 570 mV and *i*_pa_/*i*_pc_ = 0.67). Better reversibility was obtained through the combination of CT 1 and ET (Δ*E*_p_ = 200 mV and *i*_pa_/*i*_pc_ = 0.73). However, the best peak currents and reversibility were obtained when CT 2 was combined with ET (Δ*E*_p_ = 125 mV and *i*_pa_/*i*_pc_ = 1.06). This significant improvement was probably obtained when the electrodes and conductive trails were exposed to a stronger basic solution (3 min in 5.0 M NaOH), which enhances the PLA removal rate. As a result, better electrical contact and improved electronic transfer were found. These results are consistent with those obtained by EIS. As shown in Figure S4, the electrode without treatment presented the highest resistance to charge transfer (*R*_ct_ = 380 Ω cm^2^). However, this value diminished after CT 2 (*R*_ct_ = 308 Ω cm^2^) and increased more substantially after CT 2+ ET (*R*_ct_ = 150 Ω cm^2^). Thus, the optimized treatment (CT 2 + ET) was used in further experiments.

The immersion in a strongly basic solution (5.0 M NaOH up to 3 min) was proposed by Shergill and Patel^41^ to enhance the amount of CB on the surface of Protopasta filament (preprinting saponification). The immersion of 3D-printed TEI in NaOH solutions before ET was proposed by Monago-Maraña et al. (30 min in 1.0 M NaOH)^31^ and Conrado et al. (30 min in 0.5 M NaOH).^32^ Thus, CT 2 proposed here (3 min in 5.0 M NaOH) is 10-fold faster and more effective than these works (lower Δ*E*_p_ values in the presence of ferricyanide, as shown in Table 1). It is likely that our device was stable in this 5.0 M NaOH because of the better chemical resistance of PETG.^1^ This material was chosen since the first version of MICRO-EC^3D^ (PLA substrate) promptly became weak after CT 2.

**Table 1:** Features of Some Fully Automated (Single-Step) Produced TEI-EC

Material	Printing time (min)	Postprinting treatment	Δ*E*_p_ (mV)^a^	Cost (US$)	Volume (μL)	Electrical Connection	Embedded reservoir	Stirrer	Ref.
ABS/CB-PLA	7	PhotoFenton (5 min) + ET (200 s)	240	nm^b^	100	nm^b^	No	No	^24^
ABS/CB-PLA	12	DMF (170 s) + Ultrasound (30 min) + Dry (30 min) + ET (400 s)	300	0.08	100	CC^d^	Yes	No	^25^
ABS/CNT-CB-PLA	3.7	DMF (10 min) + dry (24 h) + ET (128 s)	200	0.11	nm^b^	CC^d^	No	No	^26^
PLA/CB-PLA	9	None	nm^b^	0.08	nm^b^	CC^d^	No	Yes (nd^c^)	^27^
ABS/ABS-PLA	nm^b^	None	nm^b^	0.11	2000	CC^d^	Yes	Yes (nd^c^)	^28^
PLA/CB-PLA	330	ET (400 s)	593	3.00	≤50000	CC^d^	Yes	No	^29^
PLA/CB-PLA	39	O_2_ plasma cleaner (1 min) + ET (10 CV’s in 1 M H_2_SO_4_ for 170 s)	400	nm^b^	100	3D-P^e^	Yes	No	^30^
PLA/CB-PLA	3	CT (1 M NaOH for 30 min) + ET (50 CV’s in BRB pH 7)	700	0.01	50	CC^d^	Yes	No	^31^
PLA/CB-PLA	6	CT (0.5 M NaOH for 30 min) + ET (400 s)	237	0.15	250	FPC and FFC^f^	No	No	^32^
PLA/CB-PLA	16	Polishing (1 min) + PhotoFenton (5 min) + ET (200 s)	190	nm^b^	100	nm^b^	No	No	^33^
PETG/CB-PLA	8	CT (5 M NaOH for 3 min) + ET (400 s)	125	0.04–0.10	50–300	USB^g^	Yes	Yes	This work

a Δ ***E***_**p**_ obtained in the presence of the ferri/ferrocyanide redox probe.

b **nm**: not mentioned.

c **nd**: not detailed.

d **CC**: crocodile clip (cable).

e **3D-P** (3D-printed connector): compression spring coupled to spring-loaded contact pins (cable).

f **FPC**: flexible printed circuits (cable); FFC: flexible flat cable.

g **USB**: universal serial bus; USB-SPE (wireless); USB-banana pin (cable); and USB-CC (cable).

Figure 3 illustrates the SEM images of the hybrid device containing 3D-printed PETG and CB-PLA without treatment (WT) and after each optimized treatment (CT 2 and CT 2 + ET). As shown in Figure 3A, the morphologies of the untreated PETG and CB-PLA layers were irregular with polymer excess. This explains the absence of current peaks in the CV (Figure 2, black line). However, 5.0 M NaOH (CT 2) attacked both materials, with polymer layers partially removed (Figure 3B). The PETG surface was smoother and had visible printing lines, whereas the CB-PLA surface had a rougher appearance owing to the partial removal of PLA from the surface (electrode and tracks) due to the saponification reaction and the consequent increase of the exposure of the black carbon conductive sites (increased surface porosity).^40^ This explains the observed faradaic peaks in the CV (Figure 2, green line). Furthermore, the interface of the two materials remained intact even after the reaction with the 5.0 M NaOH (without holes or cracks). After the combination of CT 2 + ET (Figure 3C), the edges of PETG and CB-PLA were intensely differentiated, resulting in less insulating material on the CB-PLA and more homogeneous surfaces. Moreover, this ET generates functional groups on the surface of CB-PLA, which enhances the electronic transfer rate.^13,40^ These findings may explain the best reversibility after the combination of CT 2 and ET (Figure 2, blue line).

**Figure 3:**
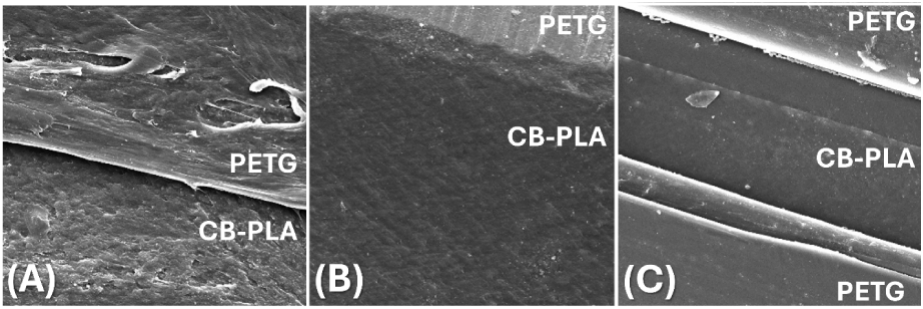
SEM images (magnification: 1000×) of cross sections from MICRO-EC^3D^ before CT 2 (A), after CT 2 (B), and after CT 2 + ET (C).

The effect of the scan rate on the CV response of MICRO-EC^3D^ in the presence of the ferricyanide redox probe was evaluated from 10 to 100 mV s^–1^ (Figure S5). As shown, the good correlation of the peak currents (*i*_pa_ and *i*_pc_) with the scan rate square root (*R* ^2^ > 0.994) indicated that the mass transfer is a diffusion-controlled process, a behavior similar to that of conventional electrodes. Replacing the slope of the curve *i*_pc_ *vs v* ^1/2^ (Figure S5) in the Randles–Sevcík equation and using the values for Fe­(CN)_6_^3–^ (*n* = 1, D = 7.7 × 10^–6^ cm^2^ s^–1^ in 0.5 M KCl), the electrochemical active surface area (ECSA) was calculated to be 0.1347 cm^2^. Assuming that the geometric area (GA) is 0.1256 cm^2^ (Ø_WE_ = 4 mm), the roughness factor (ratio between the ECSA and GA) is approximately 1.07, which suggests that the 3D-printed WE exhibits a slightly defective surface. The obtained roughness factor is similar to that reported for other CB-PLA electrodes electrochemically activated in 0.5 M NaOH.^19,32^ From this experiment and using the Nicholson method^42^ and Lavagnini equation^43^ (details in Figure S6), the heterogeneous electron transfer rate constant (*k* ^0^) was 1.5 × 10^–3^ cm s^–1^.

The interdevice reproducibility was investigated by obtaining CV curves in the presence of 1.0 mM ferricyanide and 0.5 M KCl using three MICRO-EC^3D^ fabricated using three different printing temperatures of CB-PLA (200, 210, and 220 °C). From these experiments (Table S1), good precision was obtained at all temperatures (RSD < 5%; *n* = 3), which reinforces the reproducibility of automated manufacture. However, the interelectrode precision and reversibility at 220 °C were better than those obtained at lower temperatures (200 and 210 °C). Intradevice reproducibility (repeatability) was evaluated by performing five CVs using the same device, whereas the interday reproducibility was checked through CVs over five consecutive days. For repeatability (Figure S7), all the parameters showed small variations in successive measurements (RSD < 3%; *n* = 5). Considering interday reproducibility, negligible variations in *E*_pa_ and Δ*E*_p_ and small decays in *i*_pa_ and *i*_pa_/*i*_pc_ were observed (25 and 18%, respectively). Probably, these decreases in electrochemical responses were noted due to water ingress on 3D-printed CB-PLA.^44^ The stability of MICRO-EC^3D^ was evaluated by CV (50 scans) in 5.0 mM ferricyanide at different supporting electrolytes: 0.1 M H_2_SO_4_, 0.5 M KCl, and 0.1 M NaOH (Figure S8). The RSD (1st to 50th scan) of *i*_pa_ in acidic, neutral, and basic solutions were 7.6, 1.3, and 4.5%, respectively, whereas the RSD of *E*_pa_ were 9.7, 6.2, and 5.0%. These small variations demonstrate suitable stability of the device in neutral, strong acidic, and basic solutions.

### Electrochemical Performance of MICRO-EC^3D^

The electrochemical performance of MICRO-EC^3D^ was evaluated by using stationary CV in the absence and presence of model analytes (ferricyanide, paracetamol, and nitrite). For comparison, the same measurements were performed using commercial carbon SPE (Figure 4). In this and the next studies, the smaller CB-PLA (left side) was painted with silver ink to act as the PRE. As shown in Figure S9, *E*_pa_ and *E*_pc_ of ferricyanide were shifted approximately 300 mV toward the positive direction when silver ink was used as the PRE in comparison to CB-PLA. This material enhances the stability of the potential during electrochemical measurements, as demonstrated in other TEI-EC fully 3D-printed.^13,14,16,17^ In our case, we noted that this improvement was more substantial in the case of organic analytes (PAR and OXM). As shown in Figure 4 and Table S2, MICRO-EC^3D^ presented a slightly worse reversibility than C-SPE for ferricyanide (135 vs 70 mV). Similar reversibility was obtained for paracetamol in both devices (94 vs 77 mV). For nitrite, MICRO-EC^3D^ exhibited lower peak currents than C-SPE (9.15 vs 6.03 μA). Overall, we conclude that both cells exhibited comparable electrochemical performance (*i*_p_, *E*_p_, and SD values). This is very exciting, considering that the price of MICRO-EC^3D^ is greatly lower than that of C-SPE (0.10 vs 5.00 dollars).

**Figure 4:**
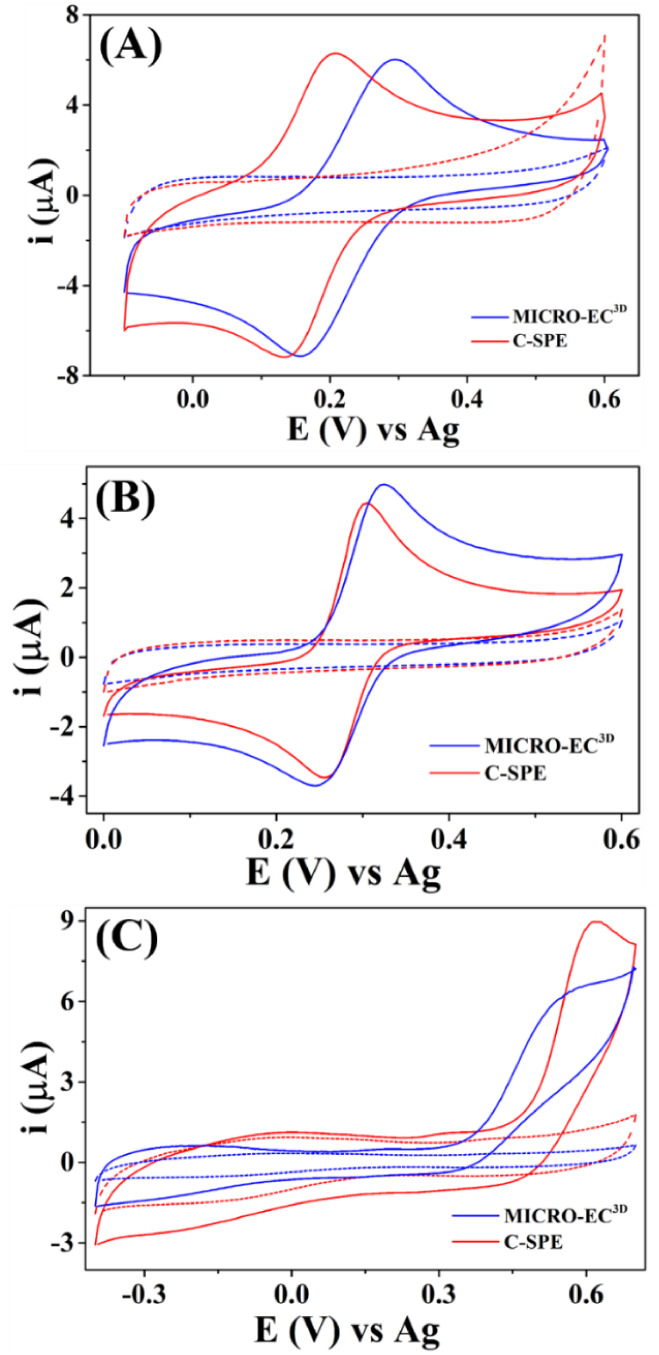
Cyclic voltammograms for MICRO-EC^3D^ (blue line) and commercial C-SPE (red line) in the absence (dashed line) and presence (solid line) of (A) 1.0 mM K_3_Fe­(CN)_6_, (B) 0.2 mM paracetamol, and (C) 0.5 mM nitrite. Electrolyte: (A) 0.5 M KCl; (B and C) 0.1 M acetate buffer (pH 5.0) and 0.5 M KCl. Scan rate: 50 mV s^–1^.

The electrochemical behavior of MICRO-EC^3D^ was also investigated under solution agitation by hydrodynamic cyclic voltammetry (HCV). For these measurements, the developed 3D-printed ministirrer was inserted into the reservoir, which contained 200 μL of analysis solution (Figure 1D and Figure S3). In HCV measurements, controlled convection (by stirring or flowing) increases the mass transport of the electroactive species toward the electrode surface, leading to higher sensitivity and reproducibility.^45^ Figure S10 presents HCV measurements in the presence of 1.0 mM ferricyanide and 0.5 M KCl. As seen, hydrodynamic profiles (steady-state current not limited by diffusion) were noted in the presence of solution convection at the three evaluated stirring speeds (slow, medium, and fast). In these cases, the cathodic limiting currents were more pronounced owing to the reduction of ferricyanide (species present in solution that feeds fresh ions to the electrode) to ferrocyanide. The cathodic limiting currents were more intense than the cathodic peak current acquired in the stationary CV. This enhancement was proportional to the stirring rate: five-, seven-, and eight-fold higher for slow, medium, and fast stirring rates, respectively. This behavior was observed because convective mass transport is significantly faster than diffusion. However, perfect hydrodynamic profiles were not obtained (higher Δ*E*_p_) in these experiments owing to the higher ohmic drop resistance of the CB-PLA electrode. While HCV has not been explored in electroanalysis, these experiments were performed to showcase the potential of the miniaturized 3D-printed set (MICRO-EC^3D^ with stirrer), which is attractive because of its portability, low cost, and requirement of small working volumes (200–300 μL). This stirrer may be used in other convective systems, such as hydrodynamic amperometry and stripping methods, to enhance the analyte preconcentration rate.

### Analytical Application of MICRO-EC^3D^

As a proof of concept, the analytical feasibility of MICRO-EC^3D^ was demonstrated for the voltammetric detection of OXM in seized samples. This analysis proves the simple screening (presumptive test) of a prohibited substance widely seized in Brazil. First, the electrochemical behavior of OXM was investigated by CV in the presence of 100 μM OXM in 0.1 M acetate buffer solution (pH 5.0). This pH value was chosen considering a single previous study for the voltammetric detection of OXM, which used a conventional three-electrode electrochemical cell (volumes in milliliter order) with a multiwalled carbon nanotube-modified glassy carbon electrode (WE), Ag/AgCl/KCl_sat._ (RE), and Pt wire (CE).^46^ As presented in Figure S11, an anodic peak was noted at around +0.7 V (vs Ag), which indicated irreversible behavior on CB-PLA. This behavior agrees with this work,^46^ which suggests that this oxidation mechanism involves the transfer of two protons and two electrons at pH values below 6.0, leading to the formation of mestanolone, the main OXM metabolite.^46^

Subsequently, an OXM was detected in the device using square-wave voltammetry (SWV) due to its better detectability and analytical speed. The SWV parameters (frequency, step potential, and pulse amplitude) were then evaluated in the univariate mode. In this study, the best compromise between detectability and peak resolution was found at a frequency of 5 Hz, a step potential of 5 mV, and a pulse amplitude of 60 mV. Using these optimized values, SW voltammograms at increasing concentrations of OXM (1.0 to 10.0 μM) were performed (Figure S12). As shown, a well-defined peak at around +0.7 V was observed for all concentrations. The peak currents increased with the OXM concentration in two linear ranges (1.0–5.0 and 6.0–10.0 μM). The linear equations were *i*_p_ (μA) = (0.050 ± 0.004) + (0.691 ± 0.019) [OXM] (μM) (*R* ^2^ = 0.996) and *i*_p_ (μA) = (2.246 ± 0.054) + (0.239 ± 0.008) [OXM] (μM) (*R* ^2^ = 0.996). This second linear range presented higher standard deviations because of the successive decrease in peak current between each replicate (*n* = 3). These results indicate saturation of the CB-PLA surface at concentrations above 6.0 μM. From the first linear range, the analytical parameters were estimated, as recommended by IUPAC guidelines.^47^ The limit of detection (LOD) and limit of quantification (LOQ) were 0.21 and 0.69 μM, respectively. The LOD was calculated as 3 × SD_B_/*S* and the LOQ as 3.3 × LOD (SD_B_ is the standard deviation of the intercept error, and *S* is the slope of the calibration curve). This LOD value is higher than that found in the previous work: 4.09 nM (1.36 ng mL^–1^).^46^ The better result obtained in the literature may be justified due to the use of a modified commercial WE and square-wave adsorptive-stripping voltammetry (SW-AdSV) for the preconcentration of the OXM on the electrode surface (deposition time: 130 s). However, the LOD obtained in MICRO-EC^3D^ by SWV (0.21 μM/69.8 ng L^–1^) is suitable for OXM detection in pharmaceutical samples (most labeled as 50 mg/tablet or capsule), which is quickly performed without the need for a previous deposition step.

Thus, the screening of OXM in two seized tablets (samples A and C) and one seized capsule (sample B) was performed on MICRO-EC^3D^. The sample solutions were prepared as described in the Experimental section (reagents and solutions), and the SW voltammograms are shown in Figure 5. As shown, the seized samples A and B exhibited a peak at around +0.7 V (blue line). These results suggest the presence of OXM in the samples (positive tests), considering that the SWV profile is quite similar to that observed for OXM (Figure S12). In contrast, sample C did not exhibit any peak, which suggests the absence of OXM in the sample (negative test). To improve our conclusions, an aliquot of the OXM standard solution was spiked into each sample solution, and new voltammograms were obtained (red lines). As shown in Figure 5, samples A and B presented increased peak currents at the same potential of the sample, which reinforces the presence of OXM in these samples (positive results). To confirm these results, the samples were analyzed by GC–MS. According to the chromatographic results, samples A and B contained OXM, and sample C did not contain OXM, which agrees with the electrochemical results. These data demonstrate the potential of MICRO-EC^3D^ as a portable and practical tool for prompt and accurate preliminary identification of OXM and other drugs in forensic samples.

**Figure 5:**
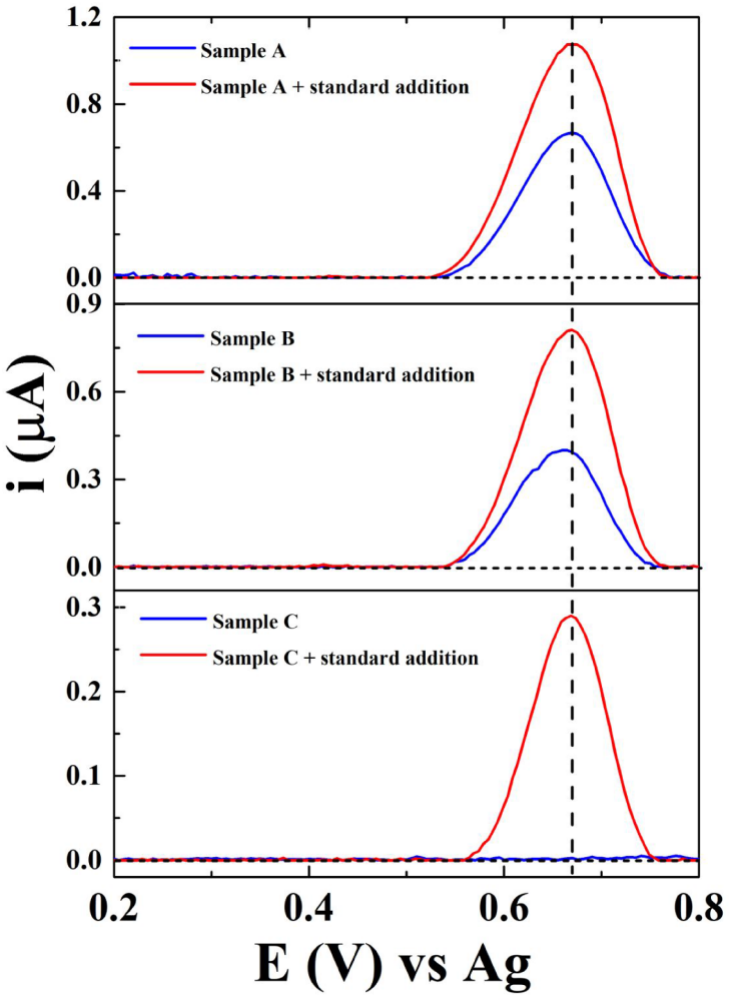
SW voltammograms recorded on MICRO-EC^3D^ for OXM screening in seized samples (A–C) before (blue lines) and after (red lines) the addition of 1.0 μM OXM standard solution. The dashed lines represent SW voltammograms of blank solutions. Conditions: Electrolyte: 0.1 M acetate buffer (pH 5.0) + 0.5 M KCl. Frequency: 5 Hz; step potential: 5 mV; pulse amplitude: 60 mV.

Finally, a search for previous analyses in samples suspected to contain OXM was conducted on drugsdata.org website, in which a sample presented methandrostenolone (another AAS) in the composition.^48^ Therefore, we conducted an interference study with this substance. As shown in Figure S13, methandrostenolone did not show electrochemical activity in the investigated potential range at MICRO-EC^3D^, indicating that this substance is not a potential interferent for OXM screening. Voltammetric detection of methandrostenolone was only reported once, in which it presented an oxidation process in a modified electrode (molecularly imprinted polymer and metal nanocomposite-modified glassy carbon electrode).^49^

It is important to emphasize that each MICRO-EC^3D^ presents limited reuse for OXM detection (mainly at higher concentrations) due to the gradual decay of peak currents between successive measurements, probably due to surface poisoning as a consequence of adsorption of oxidation products of OXM. To improve this reuse, we have tried to renew the electrode surface through polishing and novel chemical/electrochemical activation, but no success was obtained. On the other hand, a more pronounced adsorption was observed in disposable commercial SPEs, which cannot be polished for surface regeneration. Thus, although we have not been able to reactivate the MICRO-EC^3D^ surface to date, the old devices may be recycled in an extruder machine, unlike commercial SPEs that are generally discarded.

### MICRO-EC^3D^ vs Literature

The features of the proposed cell and other fully 3D-printed TEI-EC samples from the literature are presented in Table 1. As shown, MICRO-EC^3D^ combines attractive characteristics on the same device, such as portability owing to its small size, good mechanical and chemical resistance (PETG body), fast printing time (8 min) using an automated single-step production on a low-budget dual-extruder printer, low cost (0.04 and 0.10 dollars, respectively, when using CB-PLA and Ag ink as PRE), integration of a mini solution reservoir, and the use of user-friendly USB connectors. Moreover, after a relatively rapid (≈10 min) and low-toxicity activation procedure (NaOH solutions), the electrochemical responses were better than those of previous similar devices (best Δ*E*_p_ of Table 1). The embedded solution reservoir makes the device more versatile as it allows working with a range of microvolumes, from drop-scale (50 μL, suitable for qualitative analysis) to higher (150 to 300 μL, suitable for quantitative and stirring analysis). Furthermore, the developed 3D-printer stirrer is portable because it can be powered by a 5.0 V supply (e.g., a USB-port from laptops). It is important to emphasize that in the case of a commercial SPE (and other 2D-architectured TEI-EC), this variety of electrochemical systems is only possible after its use with nonportable external accessories (e.g., desktop stirrer and SPE holder). MICRO-EC^3D^ presents a simple USB wireless connection (Figure S2A), which substantially improves its portability and practicality and is especially significant for its use by inexperienced users (no training is required to perform the correct assembly of WE, CE, and RE). Although wireless connections are common in SPE, they have not been used before in 3D-printed TEI-EC to the best of our knowledge. Moreover, other user-friendly USB-wire connectors (Figure S2B and C) may be used to enable its connection with any potentiostat.

## Conclusions

In this study, a novel, fully 3D-printed electrochemical cell was proposed as a convenient tool for on-site analysis and drug screening. The device was named MICRO-EC^3D^ to highlight its small size and features: Multimaterial (PETG and CB-PLA), Integrated (body with embedded electrodes and solution reservoir), Compact (1.9 × 1.4 × 0.7 cm), Ready-to-plug (user-friendly USB connectors), and One-step printed (dual-extruder printer). This microdevice is easily assembled to a portable apparatus (wireless interface to handheld potentiostats and smartphones) and may be used under static and flowing conditions. After rapid activation protocols (CT + ET for around 10 min), the device exhibited electrochemical performance for model analytes comparable to a commercial SPE at a cost 50 times lower. However, better electrochemical responses may be obtained using more conductive filaments or upgraded activation protocols. Moreover, MICRO-EC^3D^ may be recycled and exhibits better robustness and versatility than TEI-EC produced by screen printing and laser polymer carbonization. The analytical application of the cell was successfully demonstrated for the screening of anabolic steroid oxymetholone in seized samples, and the results were consistent with those of the definitive method (GC–MS). This result demonstrates the potential use of the device in preliminary drug identification. MICRO-EC^3D^ is promising to be used in on-site and routine forensic analyses, as well as for the spread of electrochemical techniques, particularly among inexperienced users.

## Supplementary Material





## Notes

Images during and after the 3D printing of five MICRO-EC^3D^ (Figure S1); images of three MICRO-EC^3D^ USB-based connectors: wireless, banana pin, and crocodile clip (Figure S2); image of the lab-made portable stirrer set and paddle inserted in the solution reservoir of MICRO-EC^3D^ (Figure S3); Nyquist plots acquired in 3D-printed CB-PLA before and after optimized treatments (Figure S4); cyclic voltammetric scans on activated MICRO-EC^3D^ in ferricyanide at different scan rates and the relationship between *i*_p_ vs ν^1/2^ (Figure S5); plot of ψ vs. 57.75 v^–1/2^ to calculate the heterogeneous electron transfer rate constant (*k* ^0^) by the Nicholson method and Lavagnini equation (Figure S6); reversibility parameters obtained by cyclic voltammetry (*n* = 3) for CB-PLA printed at three different temperatures (Table S1); cyclic voltammetric parameters obtained in MICRO-EC^3D^ during 5 days (Figure S7); stability study of the device by successive cyclic voltammetric scans (*n* = 50) in acid, neutral, and basic electrolytes (Figure S8); cyclic voltammetric scans (blank and ferricyanide) using distinct pseudoreference electrodes at the microcell: CB-PLA or silver ink (Figure S9); comparative performance (MICRO-EC^3D^ vs commercial SPE) obtained by cyclic voltammetry in ferricyanide, paracetamol, and nitrite (Table S2); hydrodynamic cyclic voltammetry (ferricyanide on MICRO-EC^3D^) using the mini lab-made stirrer at three stirring rates (Figure S10); cyclic voltammograms in the absence or presence of oxymetholone and the proposed oxidation mechanism (Figure S11); square wave voltammograms for different oxymetholone concentrations and respective calibration plots (Figure S12); square wave voltammograms in methandrostenolone and oxymetholone solution (Figure S13) (PDF)
Speed up printing operation of one device (Video S1) (MP4)
