# Supplementary material for: MICRO (Multimaterial, Integrated, Compact, Ready-to-Plug, One-Step 3D-Printed): A Simple-to-Use Electrochemical Device for On-Site Analysis and Drug Screening
Source: ACS Omega. 2026 Feb 16;11(8):13074–83. doi: 10.1021/acsomega.5c06692 (PMC12961503; doi:10.1021/acsomega.5c06692)
Supplement: Supplementary file 1 [file ao5c06692_si_001.pdf]

Supporting Information:

MICRO (Multi-material, Integrated, Compact, Ready-to-plug, One-step 3D-printed): A simple-to-use electrochemical device for on-site analysis and drug screening

Tiago Moraes Zavarize[a], Fárlon Felipe Silva Xavier[a], Augusto dos Santos Novais[a], Celso Luciano de Araújo[a], Edmar Isaias de Melo[b], Ettore Ferrari Júnior[c], Bruno Gabriel Lucca[d], Lucas Franco Ferreira[e], Rodrigo Amorim Bezerra da Silva[a]*

[a] Institute of Chemistry, Federal University of Uberlândia, Uberlândia, 38400-902, Minas Gerais, Brazil.

[b] Institute of Chemistry, Federal University of Uberlândia, Monte Carmelo, 38500-000, Minas Gerais, Brazil.

[c] Forensic Analysis Laboratory, Criminalistic Institute, Civil Police of the Federal District. Brasília, 70610-907, Federal District, Brazil.

[d] Institute of Chemistry, Federal University of Mato Grosso do Sul, Campo Grande, 79074-460, Mato Grosso do Sul, Brazil.

[e] Institute of Science and Technology, Federal University of the Jequitinhonha and Mucuri Valleys, Diamantina, 39100-000, Minas Gerais, Brazil.

*rabsilva@ufu.br

Phone: 55-34-3810-1099

Fax: 55-34-3239-4208

Av. João Naves de Ávila, 211, CEP 38400-902, Uberlândia-MG, Brazil.

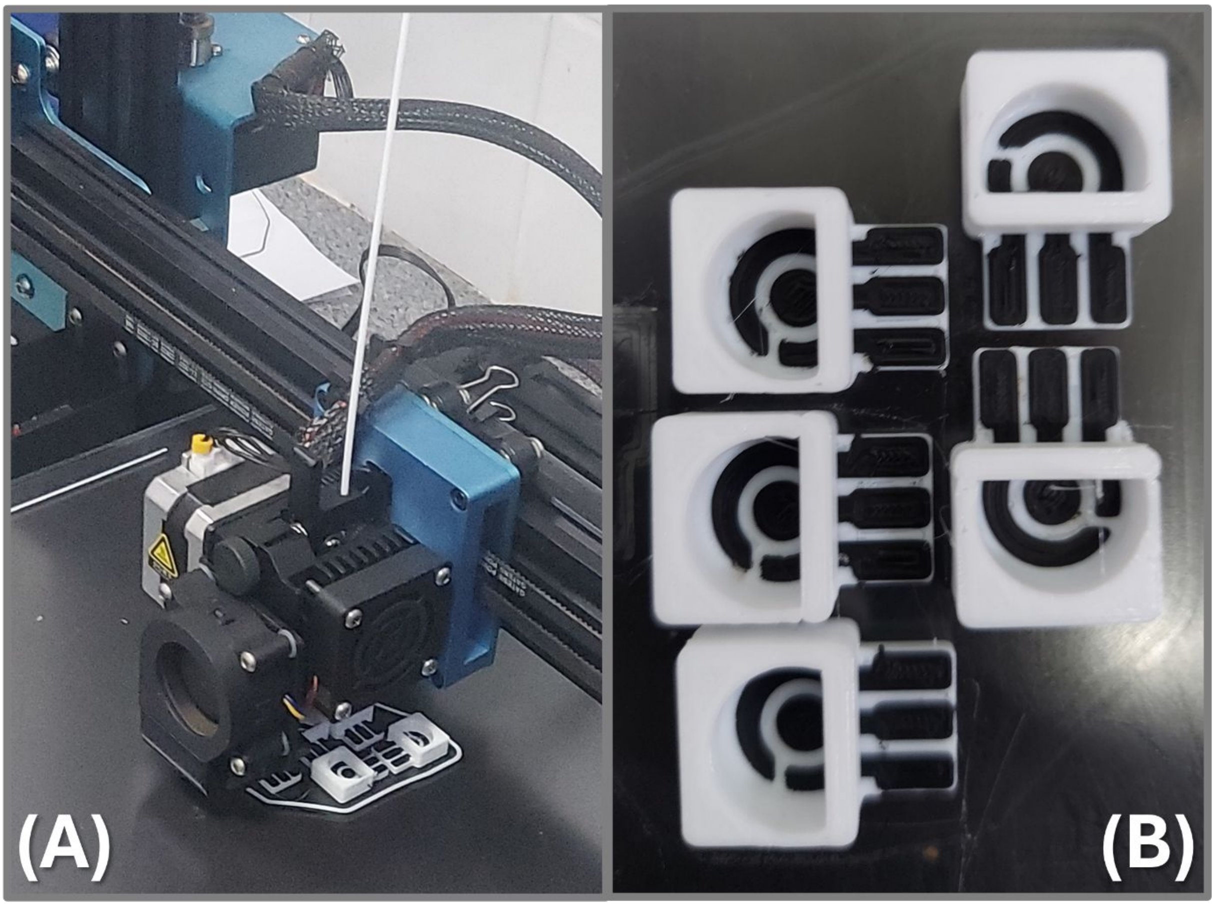

Figure S1. Images (A) during and (B) after the production of a set of five MICRO-EC^{3D}.

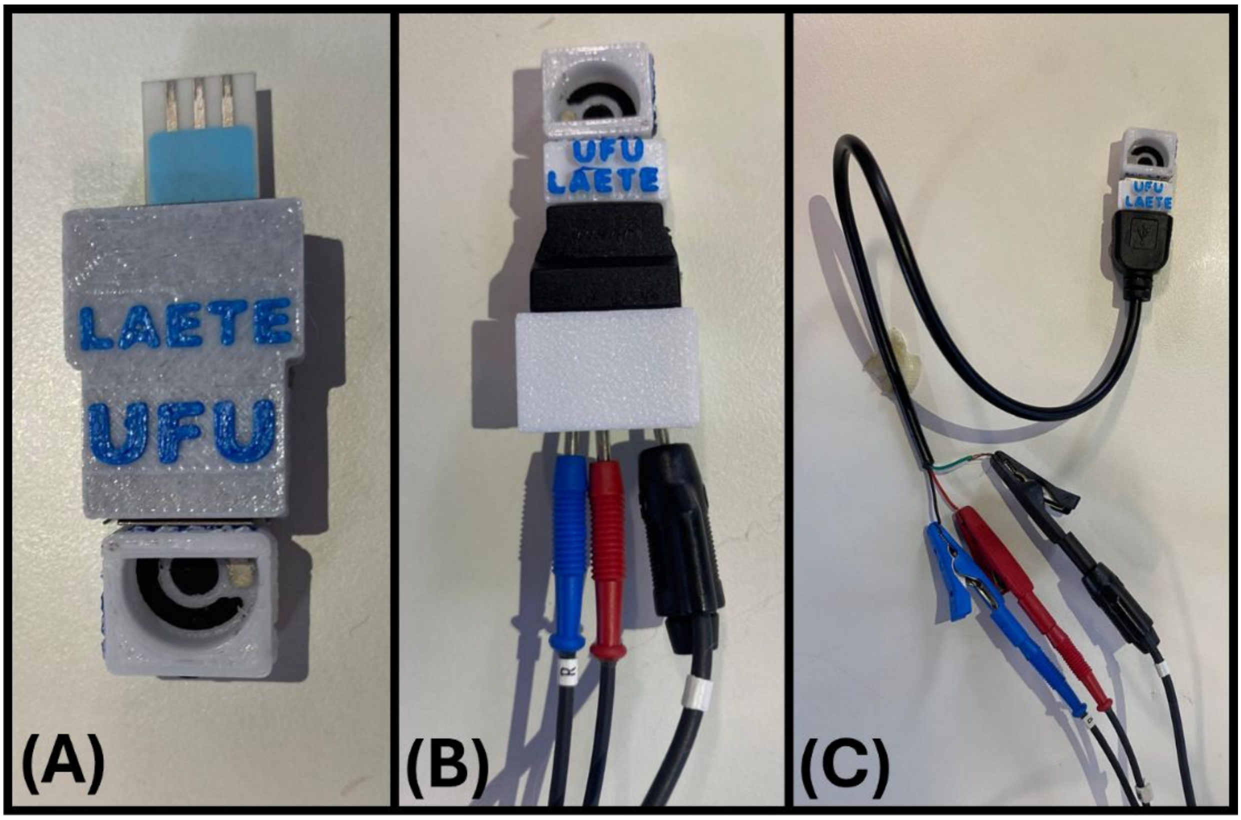

Figure S2. Image of MICRO-EC^{3D} connector (USB-type) for (A) typical SPE (Wireless/Plug and Play); (B) 2 mm banana pin, and (C) crocodile clip.

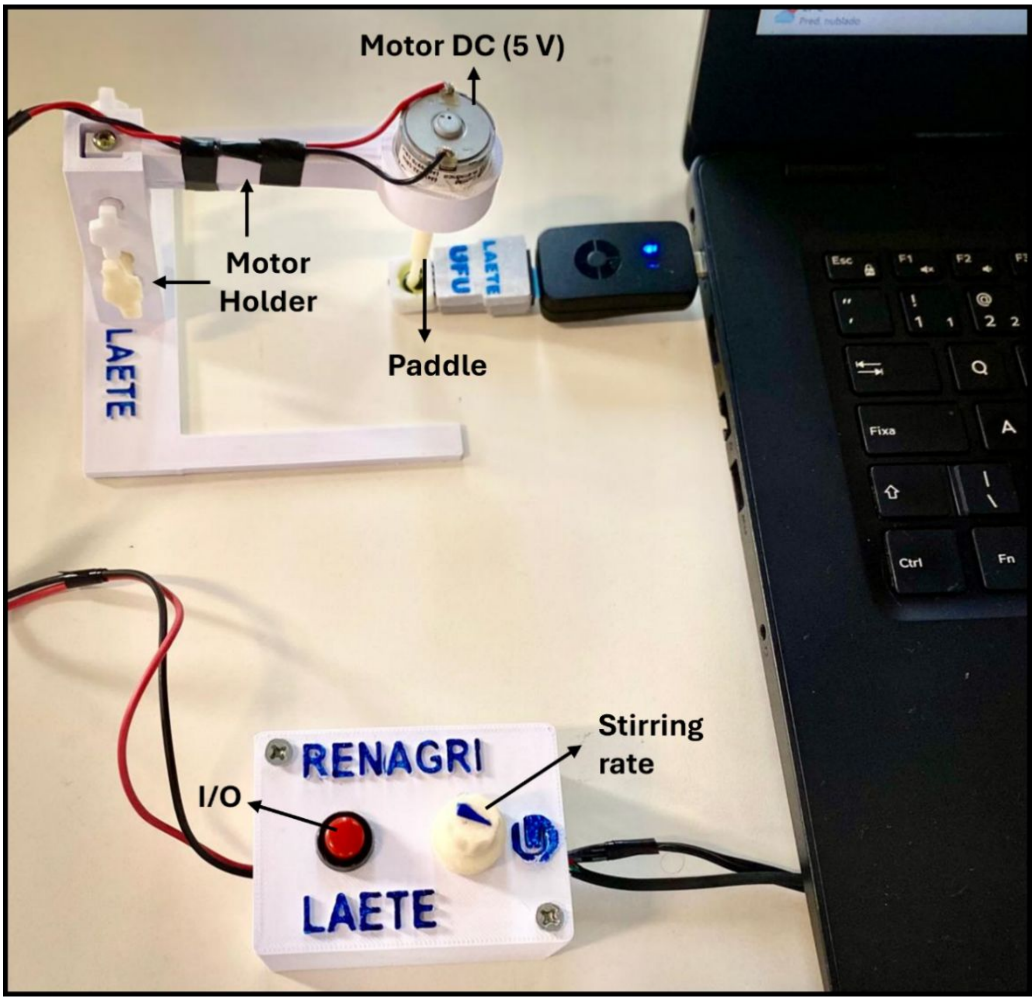

Figure S3. Picture of the components of the lab-made portable stirrer (motor, holder, paddle and controller) and MICRO-EC^{3D} connected to the miniaturized potentiostat and laptop.

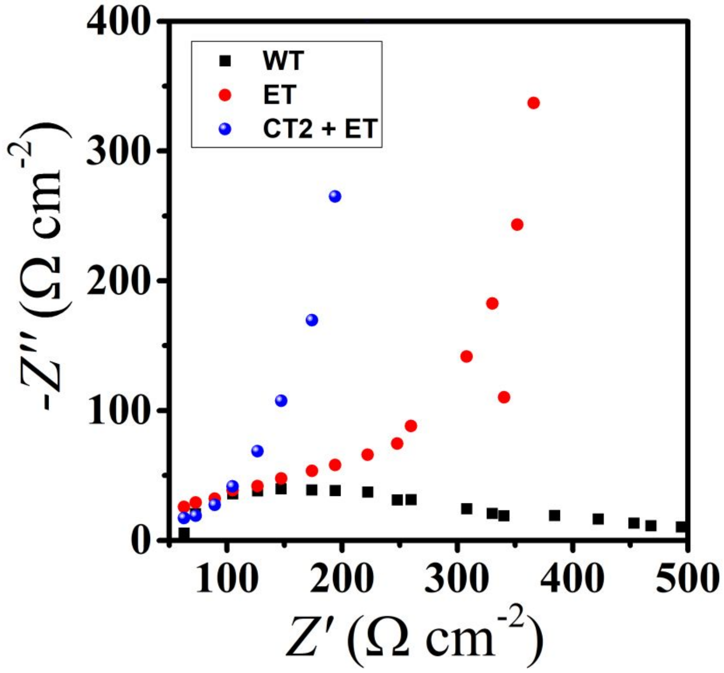

Figure S4. Nyquist plots acquired in CB-PLA without treatment (WE), after chemical treatment 2 (CT2) and electrochemical treatment (ET). EIS experiment performed in 1.0 mM Ferricyanide and 0.5 M KCl using a Frequency range between 1 Hz and 10 kHz, 10 points per decade and the half-wave potential for the applied AC voltage. Rct values were acquired using the Randles circuit.

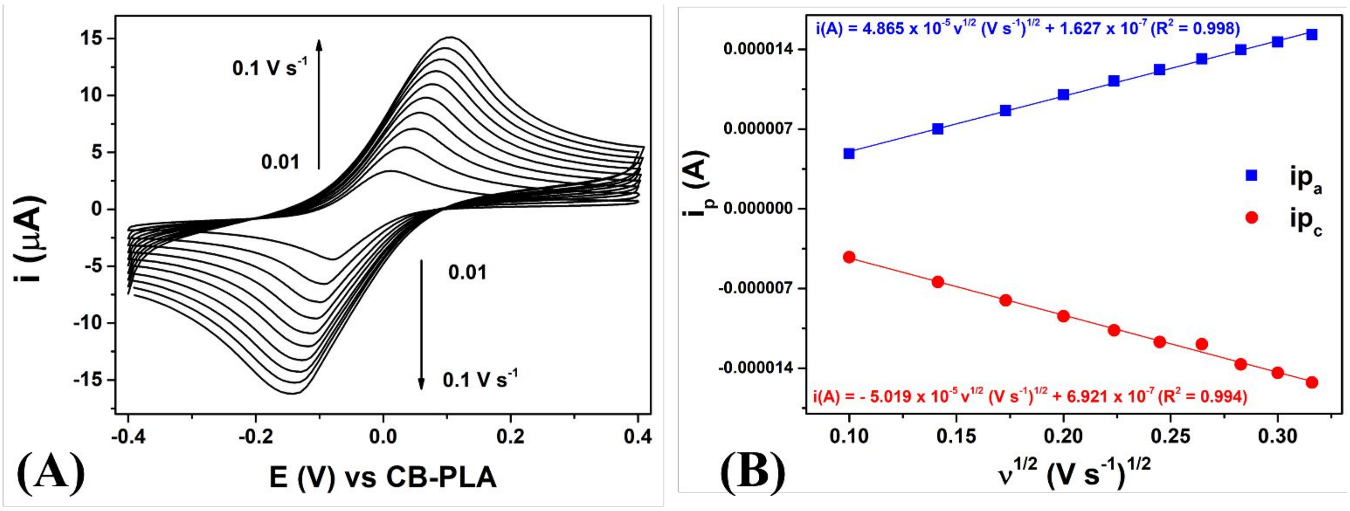

Figure S5. (A) Cyclic voltammograms obtained on MICRO-EC^{3D} in the presence of 0.5 mM ferricyanide and in 0.5 M KCl at different scan rates (10 – 100 mV s^{-1}); (B) Relationship between the i_p *vs.* $\nu^{1/2}$.

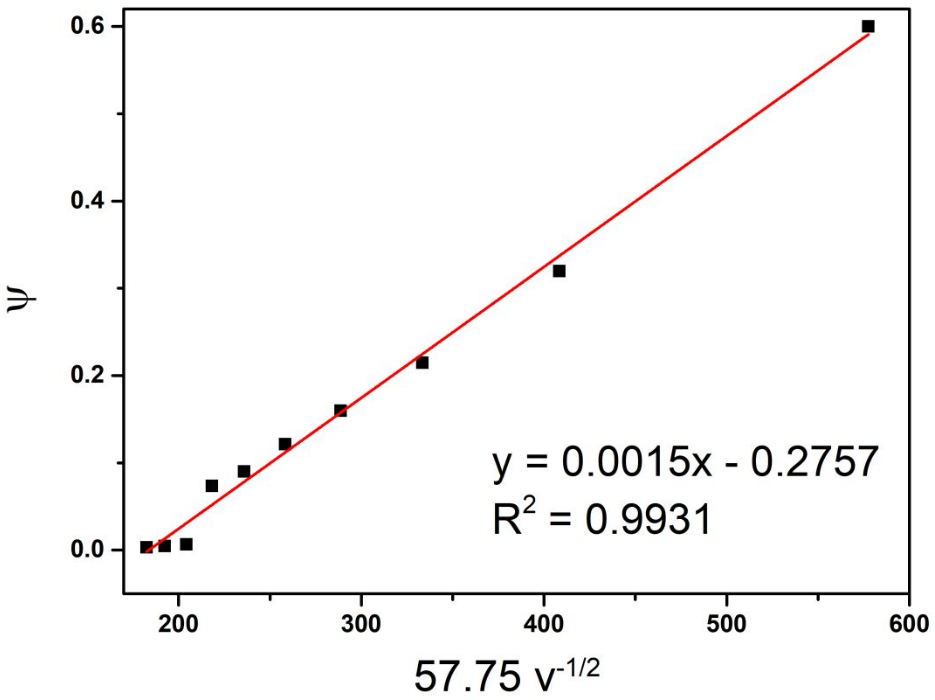

Figure S6. Plot of ψ *vs.* 57.75 $v^{-1/2}$ for the 0.5 mM ferricyanide and in 0.5 M KCl. The factor 57.75 in the abscissa represents $1/[DnF/(RT)]^{1/2}$ where D = 7.7 x 10^{-6} cm^2, n = 1, F = 96485 C mol^{-1}, R = 8.3145 J mol^{-1} K^{-1}. T = 298 K. The values of ψ were calculated using the Lavagnini equation (y = −0.6288 + 0.021 ΔE_p (mV) / (1 – 0.017 ΔE_p (mV)), in which the ΔE_p values were empirically obtained from Fig. S4. The slope of the curve corresponds to k^0 (cm s^{-1}).

Table S1. Some cyclic voltammetric parameters obtained (Mean ± SD) at three devices fabricated in the same batch using three different printing temperatures of CB-PLA (200, 210 and 220 °C; n = 3)

Printing Temperature (°C)	ΔEp (mV)	ip_a / ip_c
200	140 ± 5	0.866 ± 0.03
210	137 ± 16	0.925 ± 0.088
220	127 ± 6	0.972 ± 0.023

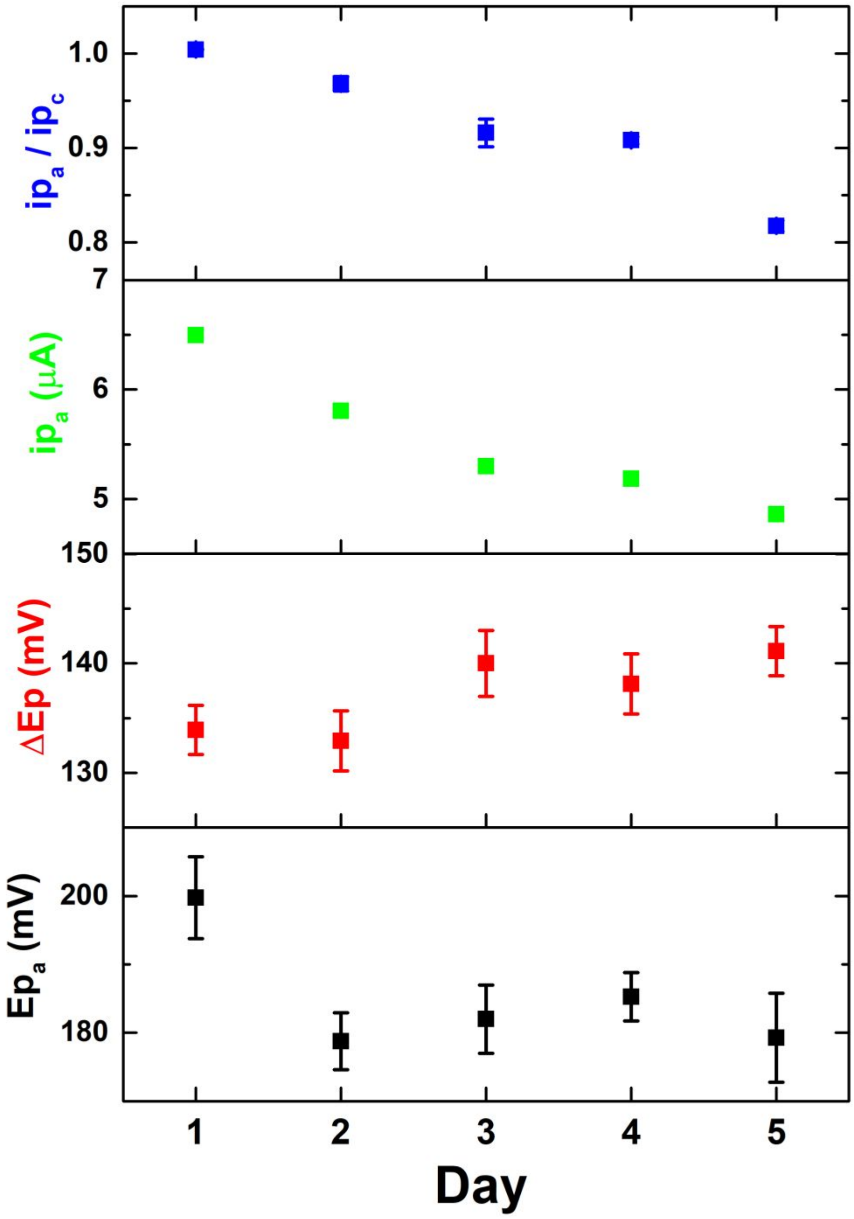

Figure S7. Electrochemical parameters obtained from five CV scans in 1.0 mM ferricyanide and 0.5 M KCl performed at the same MICRO-EC^{3D} (mean ± SD; n =5) for five days. Scan rate: 50 mV s^{-1}.

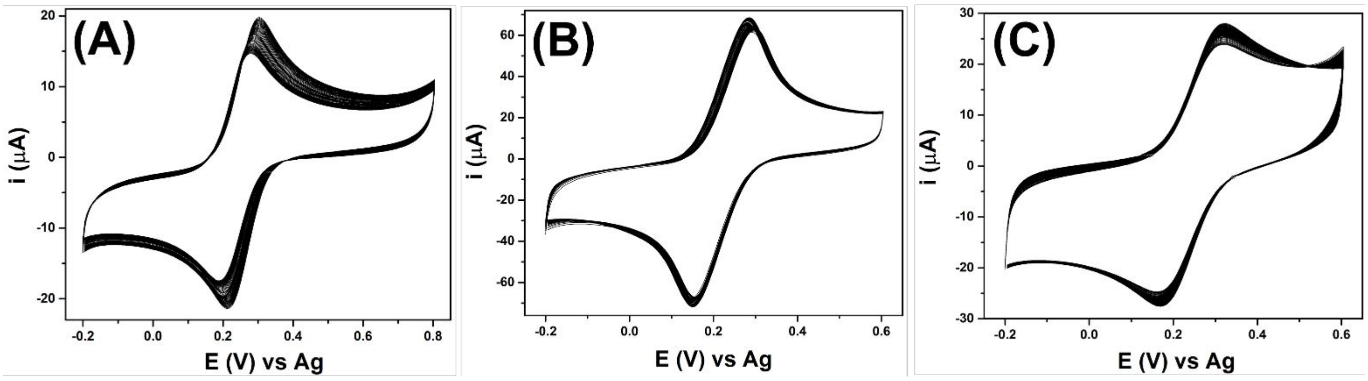

Figure S8. Successive CV scans (n = 50) of 5.0 mM ferricyanide in (A) 0.1 M H_2SO_4, (B) 0.5 M KCl and (C) 0.1 M NaOH. Scan rate: 50 mV s^{-1}.

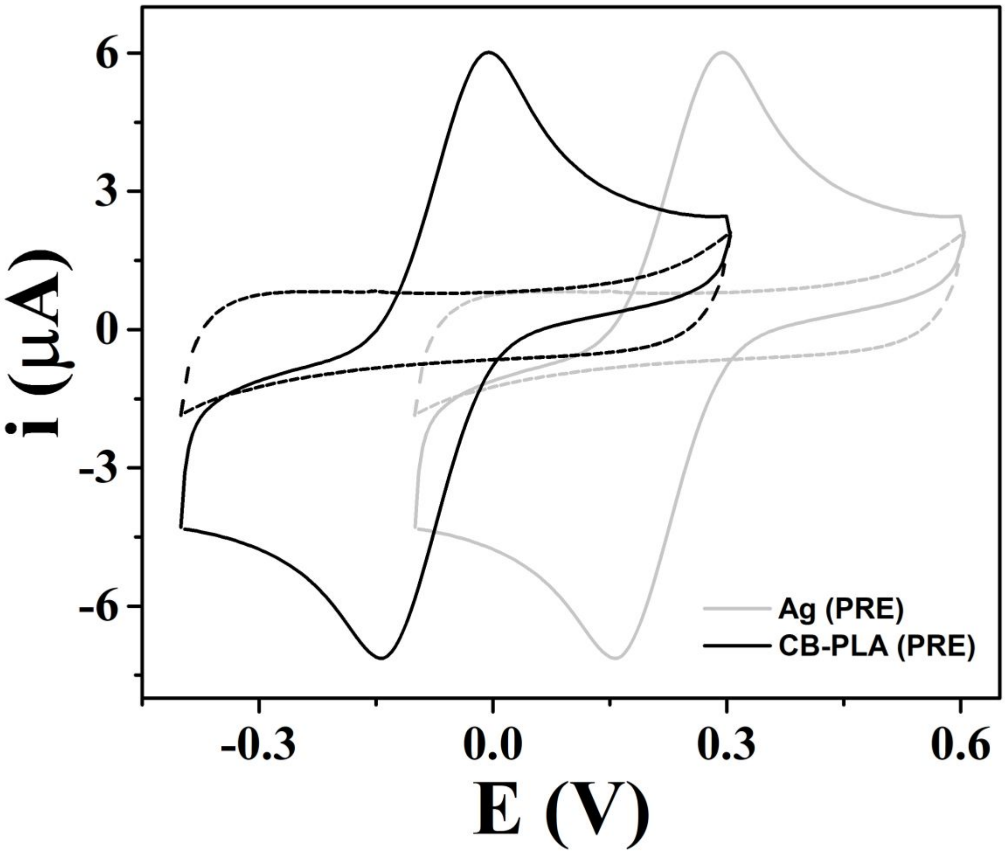

Figure S9. Cyclic voltammograms obtained on MICRO-EC^{3D} in the absence (dotted lines) and the presence (solid lines) of 1.0 mM ferricyanide using different PRE (CB-PLA or silver ink). Electrolyte: 0.5 M KCl. Scan rate: 50 mV s^{-1}.

Table S2. Electrochemical performance obtained by cyclic voltammetry on MICRO-EC^{3D} and commercial C-SPE in the presence of ferricyanide, paracetamol, and nitrite (Mean ± SD; n = 10).

	MICRO-EC^{3D}			C-SPE		
**Parameter**	**Ferricyanide**	**Paracetamol**	**Nitrite**	**Ferricyanide**	**Paracetamol**	**Nitrite**
E_{pa} (mV)	295 (5)	213 (6)	583 (6)	203 (4)	322 (2)	617 (4)
E_{pc} (mV)	160 (2)	119 (7)	-	133 (10)	245 (2)	-
ΔE_p (mV)	135 (3)	94 (6)	-	70 (10)	77 (2)	-
i_{pa} (μA)	5.27 (0.06)	4.82 (0.12)	6.03 (1)	4.98 (0.38)	4.49 (0.05)	9.15 (1)
i_{pc} (μA)	−5.57 (0.04)	−3.66 (0.03)	-	−5.23 (0.24)	−3.44 (0.01)	-
i_{pa}/i_{pc}	0.95 (0.03)	1.32 (0.06)	-	0.95 (0.30)	1.31 (0.05)	-

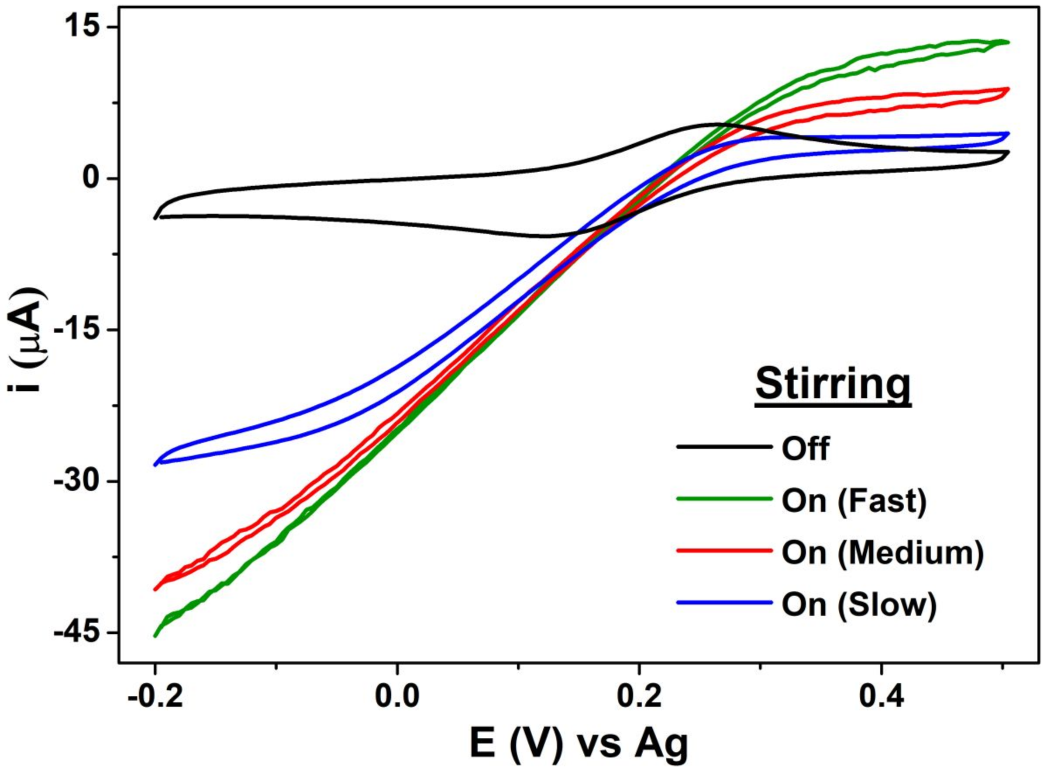

Figure S10. Stationary (stirring off) and hydrodynamic cyclic voltammograms at three stirring rates (stirring on at slow, medium and fast) in the presence of 1.0 mM $Fe(CN)_6^{3-}$. Electrolyte: 0.5 M KCl. v = 50 mV s^{-1}.

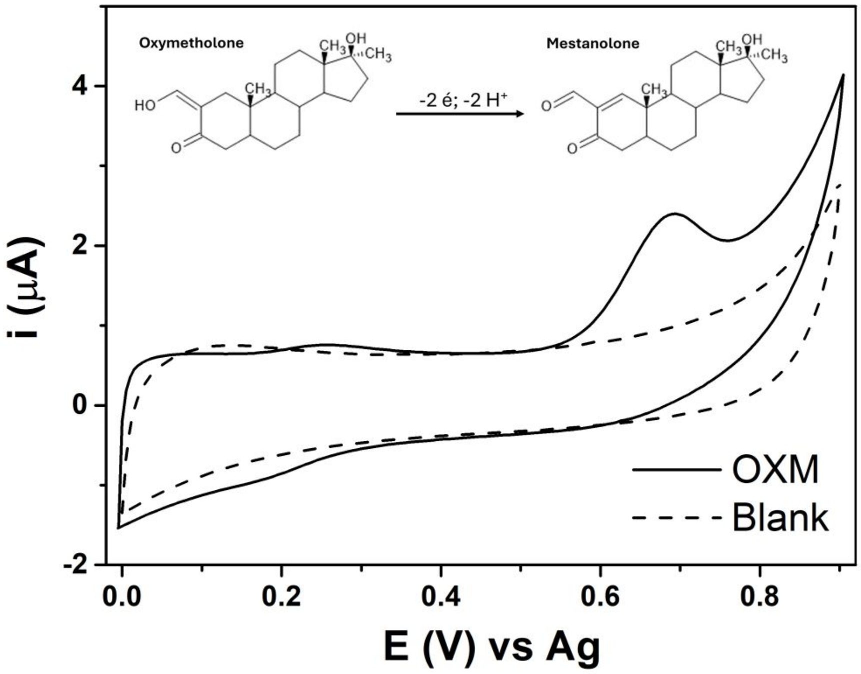

Figure S11. Cyclic voltammograms on MICRO-EC^{3D} in the absence (dashed line) and presence (solid line) of 100 μM OXM and proposed oxidation mechanism (inset).[37] Electrolyte: 0.1 M acetate buffer (pH 5.0) + 0.5 M KCl. ν = 50 mV s^{-1}.

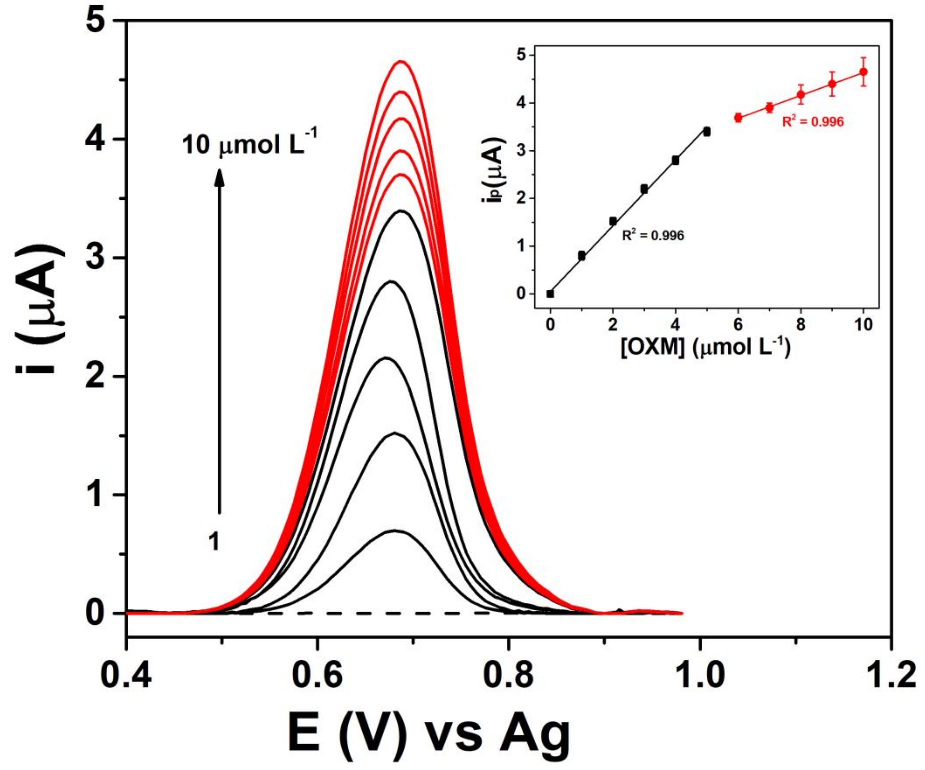

Figure S12. SW voltammograms (baseline correction) on MICRO-EC^{3D} for increasing OXM concentrations (1 – 10 μM). The inset shows the respective calibration plots (average ± SD, n = 3). Electrolyte: 0.1 M acetate buffer (pH 5.0) + 0.5 M KCl. Frequency: 5 Hz; Step potential: 5 mV; Pulse amplitude: 60 mV.

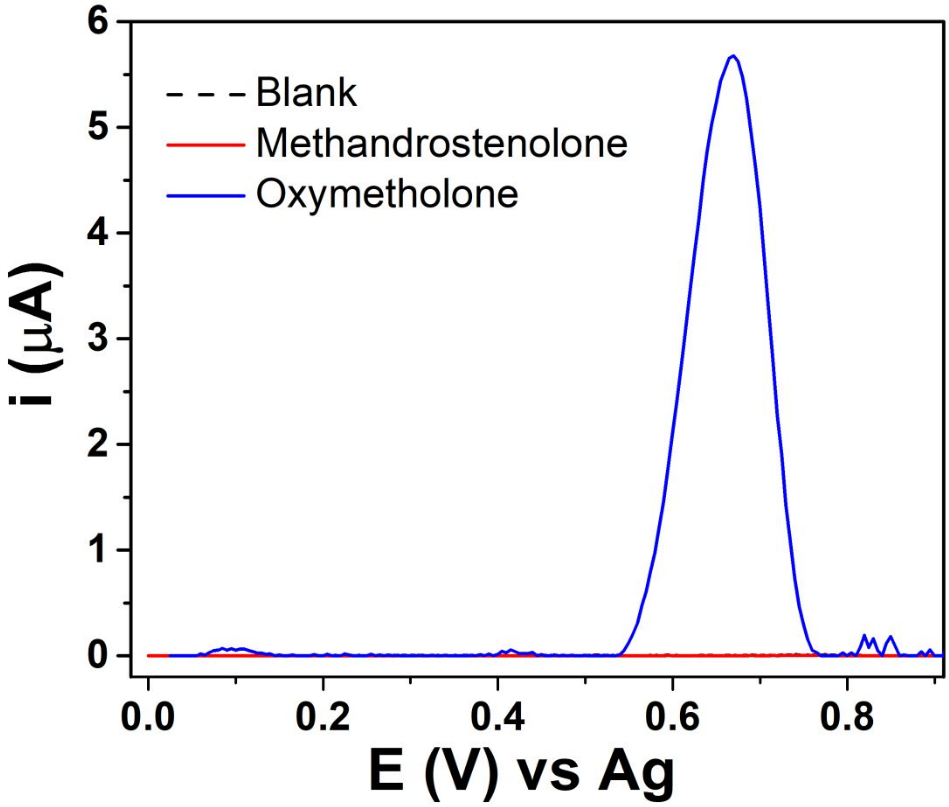

Figure S13. SWVs (baseline correction) at the MICRO-EC^{3D} in absence and presence of 15 μmol L^{-1} of methandrostenolone and oxymetholone. Other experimental conditions are the same of Fig. S12.
